# Combined treatment with caffeic and ferulic acid from *Baccharis uncinella* C. DC. (Asteraceae) protects against metabolic syndrome in mice

**DOI:** 10.1590/1414-431X20155003

**Published:** 2016-02-02

**Authors:** B.M. Bocco, G.W. Fernandes, F.B. Lorena, R.M. Cysneiros, M.A. Christoffolete, S.S. Grecco, C.L. Lancellotti, P. Romoff, J.H.G. Lago, A.C. Bianco, M.O. Ribeiro

**Affiliations:** 1 Programa de Pós-Graduação em Distúrbios do Desenvolvimento, Centro de Ciências Biológicas e da Saúde, Universidade Presbiteriana Mackenzie, São Paulo, SP, Brasil; 2 Departmento de Medicina Translacional, Escola Paulista de Medicina, Universidade Federal de São Paulo, São Paulo, SP, Brasil; 3 Centro de Ciências Naturais e Humanas, Universidade Federal de ABC, São Paulo, SP, Brasil; 4 Departmento de Ciências Patológicas da Escola de Ciências Médicas, Santa Casa, São Paulo, SP, Brasil; 5 Escola de Engenharia, Universidade Presbiteriana Mackenzie, São Paulo, SP, Brasil; 6 Instituto de Ciências Ambientais, Químicas e Farmacêuticas, Universidade Federal de São Paulo, São Paulo, SP, Brasil; 7 Department of Internal Medicine, Division of Endocrinology and Metabolism, Rush University and Medical Center, Chicago, IL, USA

**Keywords:** Obesity, Caffeic acid, Ferulic acid, Metabolic syndrome

## Abstract

Fractionation of the EtOH extract from aerial parts of *Baccharis uncinella* C. DC. (Asteraceae) led to isolation of caffeic and ferulic acids, which were identified from spectroscopic and spectrometric evidence. These compounds exhibit antioxidant and anti-inflammatory properties and have been shown to be effective in the prevention/treatment of metabolic syndrome. This study investigated whether the combined treatment of caffeic and ferulic acids exhibits a more significant beneficial effect in a mouse model with metabolic syndrome. The combination treatment with caffeic and ferulic acids was tested for 60 days in C57 mice kept on a high-fat (40%) diet. The data obtained indicated that treatment with caffeic and ferulic acids prevented gain in body weight induced by the high-fat diet and improved hyperglycemia, hypercholesterolemia and hypertriglyceridemia. The expression of a number of metabolically relevant genes was affected in the liver of these animals, showing that caffeic and ferulic acid treatment results in increased cholesterol uptake and reduced hepatic triglyceride synthesis in the liver, which is a likely explanation for the prevention of hepatic steatosis. In conclusion, the combined treatment of caffeic and ferulic acids displayed major positive effects towards prevention of multiple aspects of the metabolic syndrome and liver steatosis in an obese mouse model.

## Introduction

Metabolic syndrome is a prothrombotic and proinflammatory state characterized by visceral obesity, insulin resistance, dyslipidemia and hypertension (1). As a result of increased synthesis and uptake of cholesterol and triglycerides from plasma as well as reduction in the export of these lipids to the circulation (2,3), patients with metabolic syndrome frequently exhibit steatosis and non-alcoholic fatty liver disease (2).

Given that metabolic syndrome affects millions of individuals worldwide, there is increased interest in the development of molecules that can mitigate the metabolic consequences of obesity and liver disease. In this regard, caffeic (CA) and ferulic (FA) acids are natural organic compounds present in large amounts in the aerial parts of *Baccharis uncinella* C. DC. (Asteraceae) (4). These compounds function as key intermediate molecules in the biosynthesis of lignin, one of the principal components of plant biomass and its residues (5), and exhibit promising beneficial effects on metabolism when used in experimental models of metabolic syndrome. For example, treatment with CA has been reported to improve hyperglycemia and hepatic steatosis in animals kept on a high calorie diet, but obesity, hypertriglyceridemia and hypercholesterolemia were not always prevented (6 7 8 9). In addition, multiple studies indicate that treatment with CA improves dyslipidemia and minimizes fasting hyperglycemia, but does not always prevent diet-induced obesity (10 11 12 13).

Both CA and FA molecules only exhibit partial beneficial effects on metabolism; therefore, we investigated whether combined treatment with CA and FA could present a therapeutic advantage in the treatment of metabolic syndrome. Our results show that this combined approach with mice kept on a high-fat diet (HFD) successfully prevents obesity, dyslipidemia and liver steatosis.

## Material and Methods

### Plant material

Aerial parts of *B. uncinella* C. DC. were collected from Campos de Jordão, São Paulo State, Brazil in 2005. Botanical identification was made by Prof. Dr. Oriana A. Fávero (UPM). A voucher specimen (number SP382050) has been deposited at the Herbario da Prefeitura Municipal de São Paulo (PMSP), São Paulo, Brazil.

### Instruments

Sephadex LH-20 (Amersham Biosciences, England) was used for column chromatographic separation while silica gel 60 PF_254_ (Merck, USA) was used for analytical TLC (0.25 mm). The ^1^H and ^13^C NMR spectra were recorded on a Bruker Ultrashield Avance II spectrometer operating at 300 and 75 MHz, respectively. Spectra were performed with CD_3_OD or DMSO-d_6_ (Tedia, Brazil) using the residual solvent peak as the internal standard. The chemical shifts (δ) are given in parts per million and coupling constants (*J*) in Hz. LRESIMS was measured with a Micromass Platform mass spectrometer, operating in negative mode. Semi-preparative high-performance liquid chromatography was performed using a Dionex Ultimate 3000 chromatograph equipped with a Luna C18 column (250×10 mm, 5 µm id; Phenomenex, USA) and a UVD-DAD detector.

### Extraction and isolation

Dried and powdered aerial parts of *B. uncinella* (400 g), popularly known as *vassoura*, were extracted using EtOH at room temperature. After solvent elimination under reduced pressure, 14 g of crude extract was obtained. Part of this material (5 g) was re-suspended in EtOAc and extracted using NaOH 4% until pH 10 was attained. The alkaline phase was made acidic (pH 1) with HCl 2% and extracted with EtOAc. After drying with Na_2_SO_4_ and concentrating under reduced pressure, the EtOAc phase (2 g) was chromatographed over Sephadex LH-20 (30×2 cm), using MeOH as the eluant. This procedure provided 42 fractions (15 mL each), which were pooled into four groups (I-IV), after TLC analysis. Part of group II (120 mg) was purified by semi-preparative high-performance liquid chromatography (MeOH:H_2_O 7:3, flow rate at 1 mL/min) to produce CA (39 mg) and FA (71 mg).

### Animals and treatment

Male 8-week-old C57/BL6 mice were kept at 24°C with a 12:12-h light:dark cycle starting at 06:00 h and housed in standard plastic cages with 5 mice per cage. Food and water was provided *ad libitum*. All procedures were approved by the local Institutional Animal Care and Use Committee (CEAU/UPM #086/08/2011), according to the International Guiding Principles for Biomedical Research Involving Animals.

Eight animals were fed with either a chow diet (1.8 kcal/g) or a high fat diet (7.52 kcal/g) (Table 1) and after 20 days, glucose tolerance, cholesterolemia and triglyceridemia were assessed to verify if the mice on HFD presented the abnormalities of metabolic syndrome. Once it was confirmed that the animals exhibited metabolic syndrome, we initiated daily subcutaneous injections of CA (0.9 mg·kg^-1^·day^-1^) combined with FA (50 mg·kg^-1^·day^-1^) for more than 40 days. Food consumption and body weight were measured daily. By the end of the protocol on the 60th day, animals were lightly anesthetized with urethane (1200 mg/kg) and killed by decapitation to obtain serum and tissue samples that were immediately snap frozen for further analyses.

**Table:**
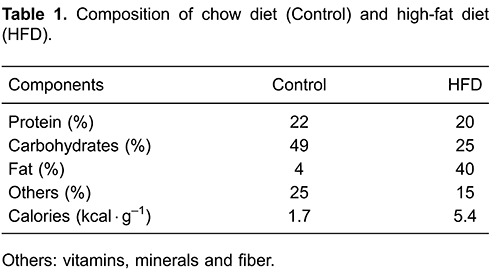

### Intraperitoneal glucose tolerance test

The animals were fasted overnight and glucose (2 g/kg) was injected intraperitoneally between 09:00 and 10:00 h. Blood samples were collected from the tail vein at the indicated times after the glucose load and glycemia were immediately determined on a glucose analyzer (LifeScan, Inc., USA).

### Blood chemistry

Total serum cholesterol and triglycerides were assessed via enzymatic methods using a commercial kit (Cholesterol Liquiform and Triglicérides Liquiform, Labtest, Brasil). Subsequently, the absorbance of the samples was measured with the NanoDrop 2000c (Thermo Scientific, USA), at wavelengths of 500 and 505 nm, respectively.

### Analysis of mRNA

The animal livers were dissected and total RNA extracted using Trizol¯ (Life Technologies Inc., USA), according to the manufacturer's instructions, and quantified by spectrophotometry (Nanodrop 2000c). For the reverse transcriptase reaction, 1.0 µg of total RNA was used in the SuperScript™ First-Strand Synthesis System for reverse transcription-polymerase chain reaction (Life Technologies Inc.) with a Mastercycler thermocycler (Eppendorf, Germany). Based on the reaction efficiency, approximately 120 ng of cDNA was used for amplification. Quantitative real time PCR was performed using QuantiTect™ SYBR^¯^ Green PCR (Qiagen, Valencia CAF with program ECO by Ilumina, USA). The cycle conditions were: 15 min at 94°C, 15 s at 94°C, 30 s at 60°C, and 30 s at 72°C for 50 cycles followed by the melting curve protocol to verify the specificity of amplicon generation. Gene expression was determined by the ΔΔCt method as described by Christoffolete (14). The housekeeping gene *GAPDH* and *beta-actin* were used as internal reference. Primer sequences are available upon request. The target genes were related to cholesterol *(LDL-R, SREBP 2, LXR, ACAT-1, HMG-CoA*), triglycerides (*SREBP-1c, ChREBP, FAS, DGAT 2, MTTP, ATGL*) and glucose (*G6pase, PPAR-α*) metabolism.

### Western blotting

Brown adipose tissue was processed for mitochondrial isolation. Mitochondrial proteins were then size-fractionated by 12% SDS-PAGE and probed with UCP1 (Santa Cruz, Biotechnology, USA) (15).

### Histology

After dissection, tissues (liver and white adipose tissue) were immersed in buffered formaldehyde solution (10%) and fixed for 24 h. Paraffin-embedded tissues were sectioned and processed as described for staining with hematoxylin-eosin or Masson's trichrome. The area of adipocytes was estimated by analysis of images photographed at 100× amplification with optical microscopy (Axioskop 2 plus, Zeiss, Germany). The images were analyzed by the program AxionVision Rel. 4.6, which estimated the area of 40 adipocytes per animal.

### Statistical analysis

The statistical analyses were done by one-way analysis of variance followed by the Student-Newman-Keuls post-test when P<0.05. For all tests, P<0.05 was considered to be statistically significant. Data are reported as means±SE.

## Results

### Characterization of caffeic and ferulic acids

**Figure 1:**
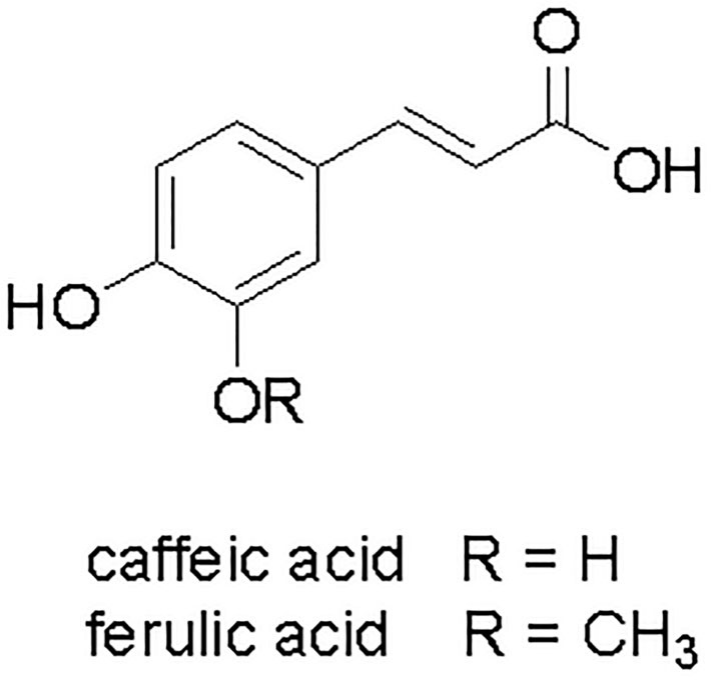
Chemical structures of caffeic acid (CA) and ferulic acid (FA), isolated from aerial parts of *B. uncinella.*

Isolated compounds from aerial parts of *B. uncinella* were characterized as derivatives of cinnamic acid by analysis of their ^1^H NMR spectra. Typical signals of the *trans*-alkene system of C_6_-C_3_ derivatives at δ 7.43/7.50 (d, *J*=16.0 Hz, H-3) and 6.25/6.30 (d, *J*=16.0 Hz, H-2), as well as multiplets of range δ 6.70-7.10, assigned to aromatic hydrogens H-5, H-8, and H-9, were observed. Additionally, the spectrum of FA was observed to have an intense peak at δ 3.67, which was assigned to one methoxyl group. The ^13^C NMR spectra of CA and FA also displayed signals ranging from δ 115 to δ 150, corresponding to aromatic ring carbon atoms (C-1 to C-6), aliphatic sp^2^ carbon atoms at δ 116 (C-8) and δ 145 (C-7), and one carboxyl group at δ 168 (C-9). With FA, an additional peak corresponding to the methoxyl group linked to C-3 was observed at δ 56.1. LRESIMS showed a deprotonated ion (M-H)^-^ peak at *m/z* 179 and 193, corresponding to the molecular formula C_9_H_8_O_4_ and C_10_H_10_O_4_ of *CA* and FA, respectively. Following analysis of our recorded data and of a description in the literature (16), identification of CA and FA was achieved (Figure 1).

### HFD and combined treatment with caffeic and ferulic acids

**Figure 2:**
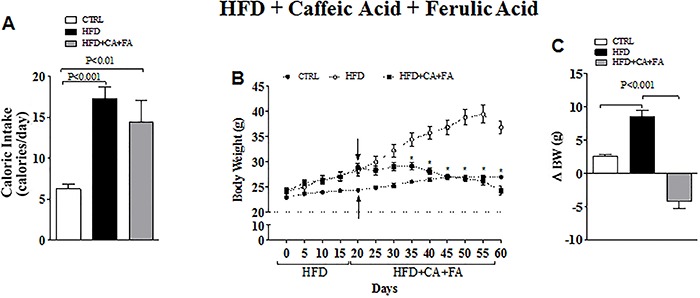
Effect of a high-fat diet (HFD) with combined treatment of caffeic acid (CA) and ferulic acid (FA) (0.9 and 50 mg·kg^-1^·day^-1^, respectively) on diet-induced obesity. *A*, Caloric intake calculated from daily food consumption. *B*, Body weight (g). Arrows indicate when the treatment with CA and FA was initiated. *C*, Body weight gain (ΔBW) after 40 days of treatment with CA and FA. Data are reported as means±SEM of five animals per group. *P<0.01 control (CTRL) *vs* HFD (ANOVA followed by the Student Newman-Keuls test).

**Figure 3:**
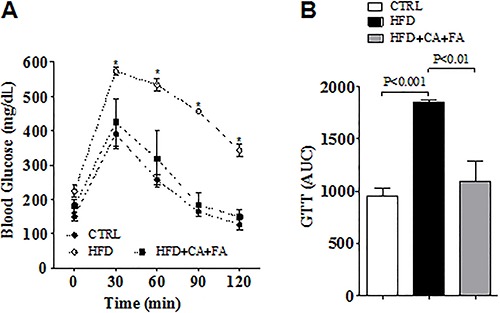
Effect of a high-fat diet (HFD) with combined treatment of caffeic acid (CA) and ferulic acid (FA) (0.9 and 50 mg·kg^-1^·day^-1^, respectively) on glucose metabolism. *A*, Blood glucose levels before and after intraperitoneal glucose tolerance test with administration of 2 g/kg glucose. *B*, Area under curve (AUC) of the glucose tolerance test (GTT) of all groups. Data are reported as means±SEM of five animals per group. *P<0.01 *vs* CTRL (ANOVA followed by the Student Newman-Keuls test).

Keeping mice on HFD for 20 days increased caloric intake (Figure 2A) and accelerated body weight gain (Figure 2B and C). The combined treatment with CA and FA was started on the 21st day of HFD after the establishment of obesity. This regimen did not affect caloric intake (Figure 2A) but it did prevent body weight gain associated with HFD. Notably, the combined treatment reduced body weight gain to levels below those observed in the Control animals (Figure 2C). In addition, the fasting hyperglycemia induced by HFD (223±17.9 *vs* Control 150±12.1 mg/dL, with P<0.05) was improved with the combined treatment with CA and FA (180±19.9 mg/dL) and normalized the glucose intolerance caused by the HFD (Figure 3).

**Figure 4:**
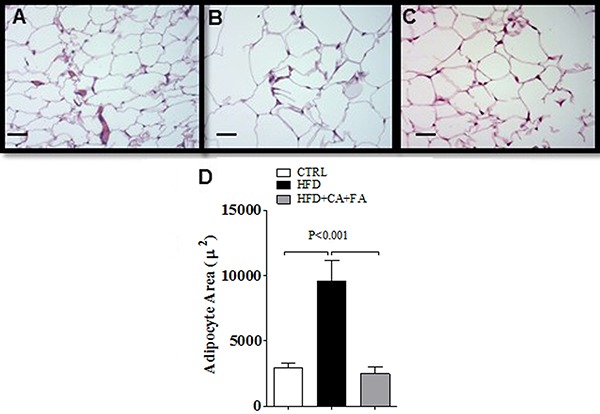
Effect of a high-fat diet (HFD) with combined treatment of caffeic acid (CA) and ferulic acid (FA) (0.9 and 50 mg·kg^-1^·day^-1^, respectively) on histology of epididymal white adipose tissue. *A,* Control (CTRL) mice; *B*, HFD treated mice; *C*, HFD+CA+FA treated mice. All magnifications are ×200. Scale bar: 50 µm. *D*, Estimated individual epididymal adipocyte area; 40 cells for each animal (five animals per group) were analyzed. ANOVA followed by the Student Newman-Keuls test were used for statistical analyses.

**Figure 5:**
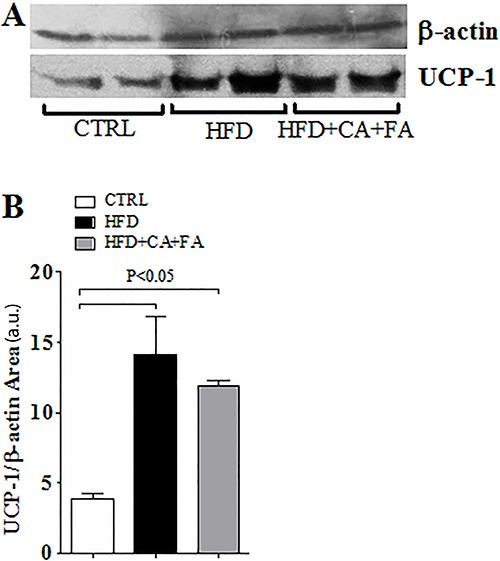
Effect of a high-fat diet (HFD) with combined treatment of caffeic acid (CA) and ferulic acid (FA) (0.9 and 50 mg·kg^-1^·day^-1^, respectively) on brown adipose tissue. *A,* UCP-1 by Western blotting of all groups; *B,* UCP-1 expression performed by Western blotting of all groups. Data are reported as means±SEM of five animals per group ANOVA followed by the Student Newman-Keuls test were used for statistical analyses.

Investigation of the epididymal adipocyte area of the animals is shown in Figure 4. Feeding on the HFD increased the adipocyte area by approximately 2.5-fold whereas combined treatment with both CA and FA prevented such increase (Figure 4D). Brown adipose tissue was also studied by assessing the UCP1 protein levels, but the elevation caused by the HFD was similar in all groups despite treatment with both CA and FA (Figure 5).

**Figure 6:**
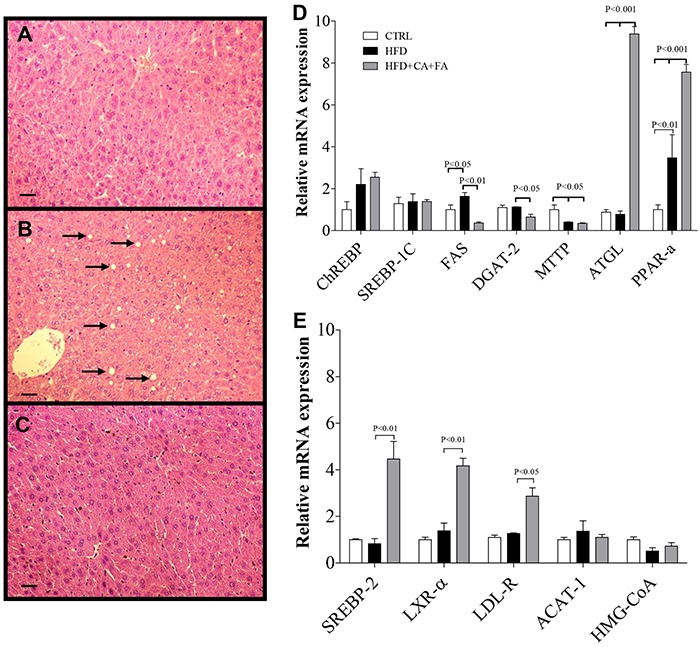
Effect of a high-fat diet (HFD) with combined treatment of caffeic acid (CA) and ferulic acid (FA) (0.9 and 50 mg·kg^-1^·day^-1^, respectively) on liver. Histology of liver stained with hematoxylin and eosin from *A*, control (CTRL) mice; *B*, HFD mice, arrows indicate the hepatic steatosis; *C*, HFD+CA+FA mice. All magnifications are ×200. Scale bar: 50 µm. *D*, Relative mRNA analysis of genes related to triglyceride metabolism: carbohydrate-responsive element-binding protein (ChREBP); sterol regulatory element-binding protein 1 (SREBP-1c); fatty acid synthase (FAS); diacylglycerol O-acyltransferase 2 (DGAT-2); microsomal triglyceride transfer protein (MTTP); adipose triglyceride lipase (ATGL); peroxisome proliferator-activated receptor alpha (PPARα). *E*, Cholesterol metabolism: sterol regulatory element-binding protein 2 (SREBP-2); liver X receptor alpha (LXR-α); low-density lipoprotein receptor (LDL-R); acetyl-CoA acetyltransferase 1 (ACAT-1); 3-hydroxy-3-methyl-glutaryl-CoA reductase (HMG-CoA). Data are reported as means±SEM of five animals per group. ANOVA followed by the Student Newman-Keuls test were used for statistical analyses.

HFD induced liver steatosis in control animals but this was prevented in the animals receiving combined treatment with CA and FA (Figure 6A-C). In addition, animals on HFD exhibited an elevation in plasma levels of cholesterol and triglycerides, but this increase was also prevented by the combined treatment with CA and FA (Table 2). These changes in liver and serum lipids were mediated by modifications in the key hepatic genes involved in lipid metabolism. Levels of FAS mRNA are decreased in animals treated with CA and FA. The gene expression for *DGAT-2*, the enzyme that catalyzes the synthesis of triglycerides, was also reduced. In addition, we also observed that the *MTTP* gene expression, the protein that transfers triglycerides to VLDL molecules, was significantly reduced by treatment with CA and FA. At the same time, the adipose triacylglycerol lipase (ATGL) mRNA levels increased with CA and FA treatment. This combined treatment also increased *PPAR-α* mRNA levels, but did not affect levels of mRNA for *ChREBP* or *SREBP-1c* mRNA (Figure 6D).

**Table:**
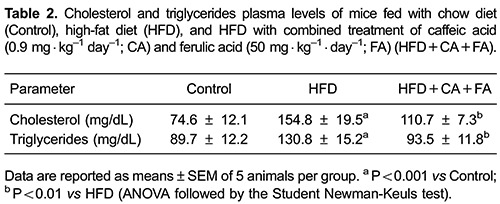

The genes involved in cholesterol metabolism were also evaluated. We found that the expression of *SREBP-2, LXR* and *LDL-R* genes all increased with the combined treatment of CA and FA. Unexpectedly, *ACAT* and *HMG-CoA* reductase mRNA levels were similar among the different groups in contrast to the data showing that cholesterol synthesis was inhibited by CA (Figure 6E).

## Discussion

The present study shows that the combination therapy with CA and FA in mice with HFD-induced metabolic syndrome prevents obesity and reverts hyperglycemia, dyslipidemia and hepatic steatosis, all conditions typically observed in mice treated with HFD only. Previous studies have shown that when used separately, CA prevented HFD-induced glucose intolerance but failed entirely in correcting obesity and dyslipidemia (6,9). Also, we found that FA alone corrected the HFD-induced dyslipidemia but failed to prevent obesity and glucose intolerance (10,11), which is in contrast to a previous report (9). In addition, studies have shown that treatment with either molecule alone prevented HFD-induced liver steatosis (6,17).

The ability of both CA and FA to prevent HFD-induced hypertriglyceridemia is remarkable and likely to involve i) a direct liver effect to coordinate reduction in the *FAS* and *DGAT-2* expressions during HFD as well as to coordinate the induction of *ATGL* and *PPARa* expression and/or ii) an indirect effect mediated via prevention of obesity. Both CA and FA have been reported to decrease FAS enzymatic activity when administered separately (8 9 10). However, it is conceivable that the beneficial effect of treatment with CA on liver steatosis is predominantly indirect, via prevention of obesity, even though treatment with CA has previously been shown to induce *PPARa* in the liver (9). Therefore, the mechanism by which CA and FA prevent HFD-induced obesity remains to be clarified. Thermogenesis in brown adipose tissue does not seem to be involved given that UCP-1 levels were not affected by treatment with both molecules. The improvement in glucose tolerance is likely to be another byproduct of obesity prevention given the known negative correlation between obesity and insulin sensitivity.

The induction of the *LDL-R, SREBP-2 and LXRa* mRNA levels in liver by combined treatment with CA and FA is striking. These changes suggest that both the uptake and export of cholesterol in the liver were increased by the action of CA and FA, resulting in increased flow of hepatic cholesterol without causing hepatic steatosis and hypercholesterolemia. This is in agreement with a previously reported *in vitro* study showing that FA facilitates the capture and degradation of LDL cholesterol by isolated hepatocytes (18). The increased expression of LXR stimulated by the treatment of CA and FA suggests increased bile acid synthesis. It is difficult to reconcile these results with previous reports that suggest treatment with CA decreases the expression of SREBP-2 protein as assessed by western blotting, which is not supported by the elevation in *LDL-R* mRNA observed in the present study. It is notable that in recent studies the combined treatment with CA and FA failed to increase mRNA levels of *HMGCoA* and *ACAT1*, especially as both molecules, when administered separately, stimulate the expression of these genes (8,9).

In conclusion, this study provides compelling experimental evidence that combination therapy with CA and FA, isolated from aerial parts of *B. uncinella* C. DC. (Asteraceae), is highly effective in preventing the multiple aspects of metabolic syndrome in a HFD mouse model. The effects of these molecules are likely to take place in the liver as evidenced by changes in the expression of key genes involved in lipid metabolism. In addition, it is likely that there are direct effects of both molecules in the adipose tissue because of their efficacy in reducing diet-induced obesity.

## References

[1] Alberti KG, Eckel RH, Grundy SM, Zimmet PZ, Cleeman JI, Donato KA (2009). Harmonizing the metabolic syndrome: a joint interim
statement of the International Diabetes Federation Task Force on Epidemiology and
Prevention; National Heart, Lung, and Blood Institute; American Heart Association;
World Heart Federation; International Atherosclerosis Society; and International
Association for the Study of Obesity. Circulation.

[2] Yki-Jarvinen H (2014). Non-alcoholic fatty liver disease as a cause and a
consequence of metabolic syndrome. Lancet Diabetes Endocrinol.

[3] Fon Tacer K, Rozman D (2011). Nonalcoholic Fatty liver disease: focus on lipoprotein
and lipid deregulation. J Lipids.

[4] Grecco SS, Felix MJ, Lago JH, Pinto EG, Tempone AG, Romoff P (2014). Anti-trypanosomal phenolic derivatives from Baccharis
uncinella. Nat Prod Commun.

[5] Boerjan W, Ralph J, Baucher M (2003). Lignin biosynthesis. Annu Rev Plant Biol.

[6] Bezerra RM, Veiga LF, Caetano AC, Rosalen PL, Amaral ME, Palanch AC (2012). Caffeic acid phenethyl ester reduces the activation of
the nuclear factor kappaB pathway by high-fat diet-induced obesity in
mice. Metabolism.

[7] Jung UJ, Lee MK, Park YB, Jeon SM, Choi MS (2006). Antihyperglycemic and antioxidant properties of caffeic
acid in db/db mice. J Pharmacol Exp Ther.

[8] Liao CC, Ou TT, Wu CH, Wang CJ (2013). Prevention of diet-induced hyperlipidemia and obesity by
caffeic acid in C57BL/6 mice through regulation of hepatic lipogenesis gene
expression. J Agric Food Chem.

[9] Cho AS, Jeon SM, Kim MJ, Yeo J, Seo KI, Choi MS (2010). Chlorogenic acid exhibits anti-obesity property and
improves lipid metabolism in high-fat diet-induced-obese mice. Food Chem Toxicol.

[10] Son MJ, Rico CW, Nam SH, Kang MY (2011). Effect of oryzanol and ferulic acid on the glucose
metabolism of mice fed with a high-fat diet. J Food Sci.

[11] Jin Son M, Rico W, Hyun Nam S, Young Kang M (2010). Influence of oryzanol and ferulic Acid on the lipid
metabolism and antioxidative status in high fat-fed mice. J Clin Biochem Nutr.

[12] Totani N, Tateishi S, Takimoto T, Shinohara R, Sasaki H (2012). Ferulic acid esters and weight-loss promoting effects in
rats. J Oleo Sci.

[13] Ardiansyah, Shirakawa H, Koseki T, Hashizume K, Komai M (2007). The Driselase-treated fraction of rice bran is a more
effective dietary factor to improve hypertension, glucose and lipid metabolism in
stroke-prone spontaneously hypertensive rats compared to ferulic
acid. Br J Nutr.

[14] Christoffolete MA, Linardi CC, de Jesus L, Ebina KN, Carvalho SD, Ribeiro MO (2004). Mice with targeted disruption of the Dio2 gene have
cold-induced overexpression of the uncoupling protein 1 gene but fail to increase
brown adipose tissue lipogenesis and adaptive thermogenesis. Diabetes.

[15] de Jesus LA, Carvalho SD, Ribeiro MO, Schneider M, Kim SW, Harney JW (2001). The type 2 iodothyronine deiodinase is essential for
adaptive thermogenesis in brown adipose tissue. J Clin Invest.

[16] Prachayasittikul S, Suphapong S, Worachartcheewan A, Lawung R, Ruchirawat S, Prachayasittikul V (2009). Bioactive metabolites from *Spilanthes
acmella* Murr. Molecules.

[17] Kesh SB, Sikder K, Manna K, Das DK, Khan A, Das N (2013). Promising role of ferulic acid, atorvastatin and their
combination in ameliorating high fat diet-induced stress in mice. Life Sci.

[18] Srinivasan M, Sudheer AR, Pillai KR, Kumar PR, Sudhakaran PR, Menon VP (2006). Influence of ferulic acid on gamma-radiation induced DNA
damage, lipid peroxidation and antioxidant status in primary culture of isolated
rat hepatocytes. Toxicology.

